# Inhibition of microRNA-711 limits angiopoietin-1 and Akt changes, tissue damage, and motor dysfunction after contusive spinal cord injury in mice

**DOI:** 10.1038/s41419-019-2079-y

**Published:** 2019-11-04

**Authors:** Boris Sabirzhanov, Jessica Matyas, Marina Coll-Miro, Laina Lijia Yu, Alan I. Faden, Bogdan A. Stoica, Junfang Wu

**Affiliations:** 1 Department of Anesthesiology and Center for Shock, Trauma and Anesthesiology Research (STAR), School of Medicine, Baltimore, MD USA; 2 University of Maryland Center to Advance Chronic Pain Research, University of Maryland Baltimore, Baltimore, MD 21201 USA

**Keywords:** Cell death in the nervous system, Trauma

## Abstract

Spinal cord injury (SCI) causes neuronal cell death and vascular damage, which contribute to neurological dysfunction. Given that many biochemical changes contribute to such secondary injury, treatment approaches have increasingly focused on combined therapies or use of multi-functional drugs. MicroRNAs (miRs) are small (20–23 nucleotide), non-protein-coding RNAs and can negatively regulate target gene expression at the post-transcriptional level. As individual miRs can potentially modulate expression of multiple relevant proteins after injury, they are attractive candidates as upstream regulators of the secondary SCI progression. In the present study we examined the role of miR-711 modulation after SCI. Levels of miR-711 were increased in injured spinal cord early after SCI, accompanied by rapid downregulation of its target angiopoietin-1 (Ang-1), an endothelial growth factor. Changes of miR-711 were also associated with downregulation of the pro-survival protein Akt (protein kinase B), another target of miR-711, with sequential activation of glycogen synthase kinase 3 and the pro-apoptotic BH3-only molecule PUMA. Central administration of a miR-711 hairpin inhibitor after SCI limited decreases of Ang-1/Akt expression and attenuated apoptotic pathways. Such treatment also reduced neuronal/axonal damage, protected microvasculature and improved motor dysfunction following SCI. In vitro, miR-711 levels were rapidly elevated by neuronal insults, but not by activated microglia and astrocytes. Together, our data suggest that post-traumatic miR-711 elevation contributes to neuronal cell death after SCI, in part by inhibiting Ang-1 and Akt pathways, and may serve as a novel therapeutic target.

## Introduction

Traumatic spinal cord injury (SCI) initiates a complex cascade of molecular events that leads to progressive degeneration. These secondary injury processes include induction of multiple neuronal cell death pathways and vascular disruption, leading to neuronal loss, axonal damage, and neurological dysfunction. The failure of effective clinical therapeutic interventions for SCI likely reflects, in part, the incomplete understanding of complex secondary injury mechanisms and the historical emphasis on targeting single injury mechanisms.

MicroRNAs (miRs) are short (20–23 nucleotide) noncoding RNA molecules that negatively regulate gene expression at the post-transcriptional level by binding to the 3′-untranslated region (UTR) of target mRNAs, leading to their degradation and/or translational inhibition^1^. As single miRs can simultaneously modulate the expression of various proteins in multiple pathways, miRs are potentially attractive upstream regulatory targets for secondary SCI. A number of miRNAs are enriched in neuronal cells and have important roles in central nervous system (CNS) development, function and pathology- including synaptic plasticity, dendritogenesis and structural remodeling^2^. Due to their ability to regulate entire networks of genes^3^, neuronal miRs can act as “meta-controllers” of CNS gene expression^4,5^. Recent studies indicate that miRs are involved in the pathophysiology of CNS ischemia and trauma^6–14^. Although miRs have been shown to modulate neuronal cell death pathways, few have been directly evaluated within the context of SCI^11–14^, and their mechanisms of action in this regard remain largely unknown. It has been suggested that concurrent inhibition of multiple pro-apoptotic molecules, such as members of the BH3-only family of pro-apoptotic genes, may underlie the strong anti-apoptotic effects of certain miRs^15–17^.

We have recently initiated a comprehensive effort to explore changes in miRs expression after mouse SCI, using microarray analyses followed by qPCR validation. Among miRs showing highly significant changes early after traumatic injury in vivo was miR-711. Previous studies have suggested that among the mRNA targets with potential sites for miR-711 in their 3′UTR are several neuroprotective molecules, including Akt (protein kinase B) and angiopoietin-1 (Ang-1)^18–20^. The intracellular Akt/glycogen synthase kinase (GSK) β signaling pathway appears to be essential for regulating axonal growth; inhibition of this pathway has proven neuroprotective and shows axon-growth-promoting effects in SCI^21–25^. Ang-1 is known to protect endothelial cell survival and also to preserve the integrity of endothelial cell tight junctions under pathological conditions^26,27^. We recently demonstrated that Ang-1 is a target for miR-711 in neuronal injury^28^. Ang-1 can limit neuronal apoptosis, an effect that involves Tie-2 (Ang-1 receptor)-mediated activation of the phosphatidylinositol 3-kinase (PI3K)/Akt pathway and inhibition of caspase-3 cleavage^29^. In addition, Ang-1 promotes axonal remodeling and neurite outgrowth in vitro and after brain ischemia^30–32^.

The goal of this study was to determine whether SCI-induced elevation of miR-711 contributes to neuronal and axonal damage through inhibition of Akt and Ang-1 signaling, as well as whether miR-711 inhibition can reduce secondary injury after SCI. We first examined the temporal expression profiles of miR-711 and its known targets in a well-established mouse contusion SCI model. MiR-711 hairpin inhibitor or negative control-treated mice were compared with assess the role of miR-711 in neuronal cell death, axonal damage and associated neurological dysfunction.

## Results

### miR-711 is upregulated in the injured spinal cord tissue

**Fig. 1:**
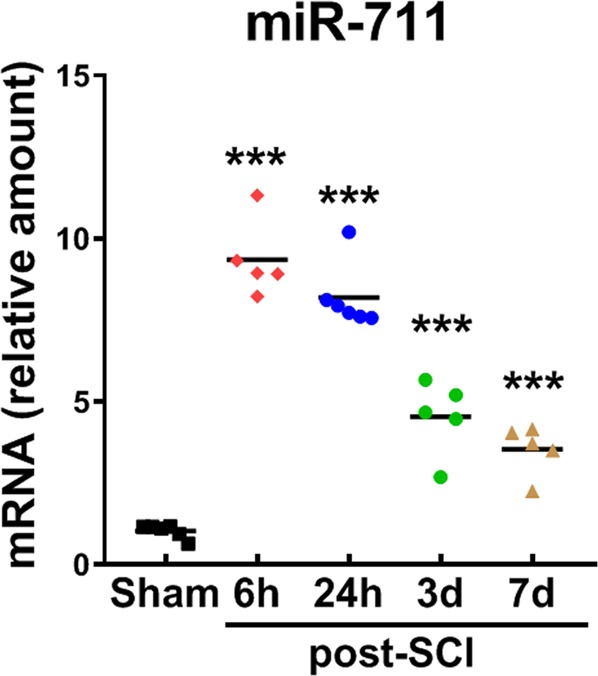
miR-711 is rapidly elevated after SCI. Spinal cord samples (~3-mm segments) were collected at indicated time points, and processed for RNA extraction and miR-711 qPCR analysis. miR-711 expression was normalized to U6 snRNA. All data are presented as independent data points. One-way ANOVA was performed followed by Newman-Keuls multiple comparisons test. ***p < 0.001 vs. Sham, *n* = 5–6 mice/group

miRNA microarray profiling by GeneChip miRNA array revealed that CNS trauma significantly upregulate the level of miR-711 in the ipsilateral cortex of controlled cortical impact mice^33^ as well as the injured spinal cord (data not shown). Here we performed a detailed expression profiling of miR-711 in the injured spinal cord after a moderate contusion SCI in mice. qPCR analysis demonstrated a rapid and persistent upregulation (9–10-folds) of miR-711 (*n* = 5–6 mice/group), starting as early as 6 h post-injury, and persisting through 7 days (Fig. 1).

### Central administration of miR-711 hairpin inhibitor increases the level of Akt and phosphorylation of its targets in the injured spinal cord

**Fig. 2:**
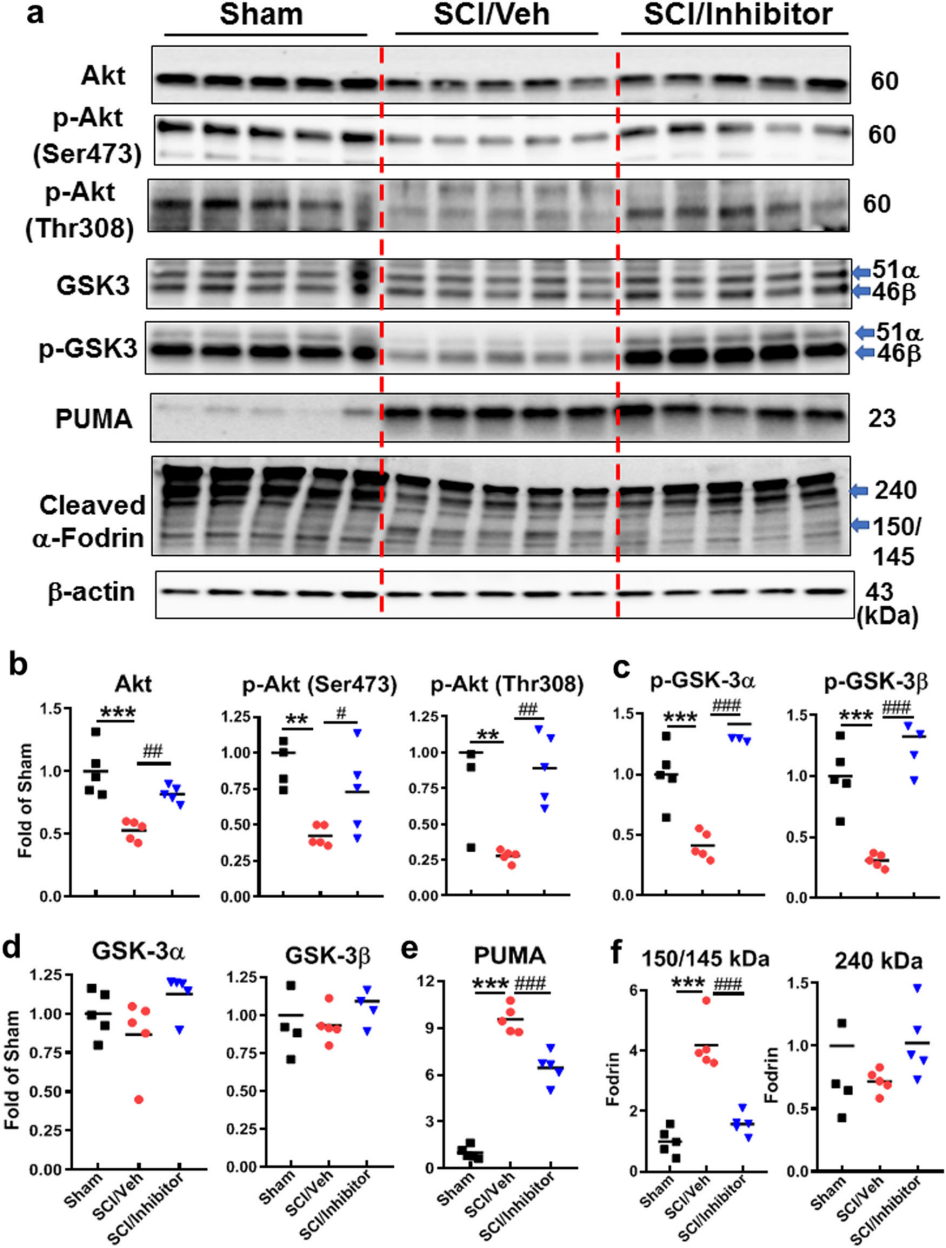
Intrathecal administration of miR-711 hairpin inhibitor increases Akt/GSK3α/β signaling and attenuates expression of PUMA and apoptotic marker fodrin cleavage at 24 h after SCI. Mice were treated with miR-711 hairpin inhibitor or negative control (vehicle) at 5 min post-injury. **a** Representative western blots for total Akt, phosphorylated Akt (p-Akt, Ser473, Thr308), total and phosphorylated GSK3α/β, PUMA, cleaved α-fodrin. **b–f** Protein levels were quantified by densitometry, normalized to β-actin, and presented as fold change compared with sham-injured controls. All data are presented as independent data points. One-way ANOVA was performed followed by Newman-Keuls multiple comparisons test. *n* = 5/group. **p < 0.01, ***p < 0.001 vs. Sham; ^#^*p* < 0.05, ^##^*p* < 0.01; ^###^*p* < 0.001 vs. SCI/Vehicle group

To assess whether miR-711 elevation is upstream of Akt pathway inhibition and BH3-only activation after SCI we administered miR-711 hairpin inhibitors or negative control after SCI (intrathecal injection, 0.5 nmol; right after injury) as previously described^34^. At 24 h after injection, western blot analysis demonstrated that SCI significantly decreased levels of Akt (*p* < 0.001, vs. Sham), general phosphorylated Akt and specifically phosphorylated Akt (*p* < 0.01, vs. Sham, Fig. 2a, b), and markedly downregulated phosphorylation of Akt substrates GSK3α/β (Fig. 2c, *p* < 0.001, vs. Sham). Total GSK3α/β levels were unaltered at 24 h after SCI (Fig. 2d). Notably, miR-711 hairpin inhibitors significantly reversed these changes and upregulated the total and activated (pSer473, pThr308) Akt (*p* < 0.01, vs. SCI/Veh), increased Akt-dependent phosphorylation (inhibition) of GSK3α/β (*n* = 5 mice/group, *p* < 0.001, vs. SCI/Veh). We did not observe differences between Sham/inhibitor and Sham/Vehicle groups (data not shown).

Next, we examined the effect of miR-711 inhibition on SCI-mediated upregulation of pro-apoptotic member of Bcl-2 family PUMA (Bcl2-binding component 3), which expression at least in part downregulated by Akt pathway. SCI resulted in a 10-fold increase of BH3-only molecules PUMA (*p* < 0.001, vs. Sham/Veh). Treatment with the miR-711 hairpin inhibitor significantly decreased injury-dependent activation of PUMA after SCI (Fig. 2e, *p* < 0.001, vs. SCI/Veh). Furthermore, cleavage of the cell death marker α-fodrin, was assessed in the injured spinal cord at 24 h post-injury. 150/145 kDa fragments of α-fodrin cleavage were increased 4–5-fold, whereas full-length protein (240 kDa) and the level of 120 kDa fragment were slightly decreased in SCI versus sham spinal cord. These changes were significantly attenuated in SCI mice treated with miR-711 inhibitor (Fig. 2f, *p* < 0.001, vs. SCI/Veh), confirming reduced cell death upon miR-711 inhibition. We observed no differences of miR-711 inhibitor in sham mice compared with Sham/Vehicle group (data not shown).

### miR-711 hairpin inhibitor confers neuroprotection in the injured spinal cord

**Fig. 3:**
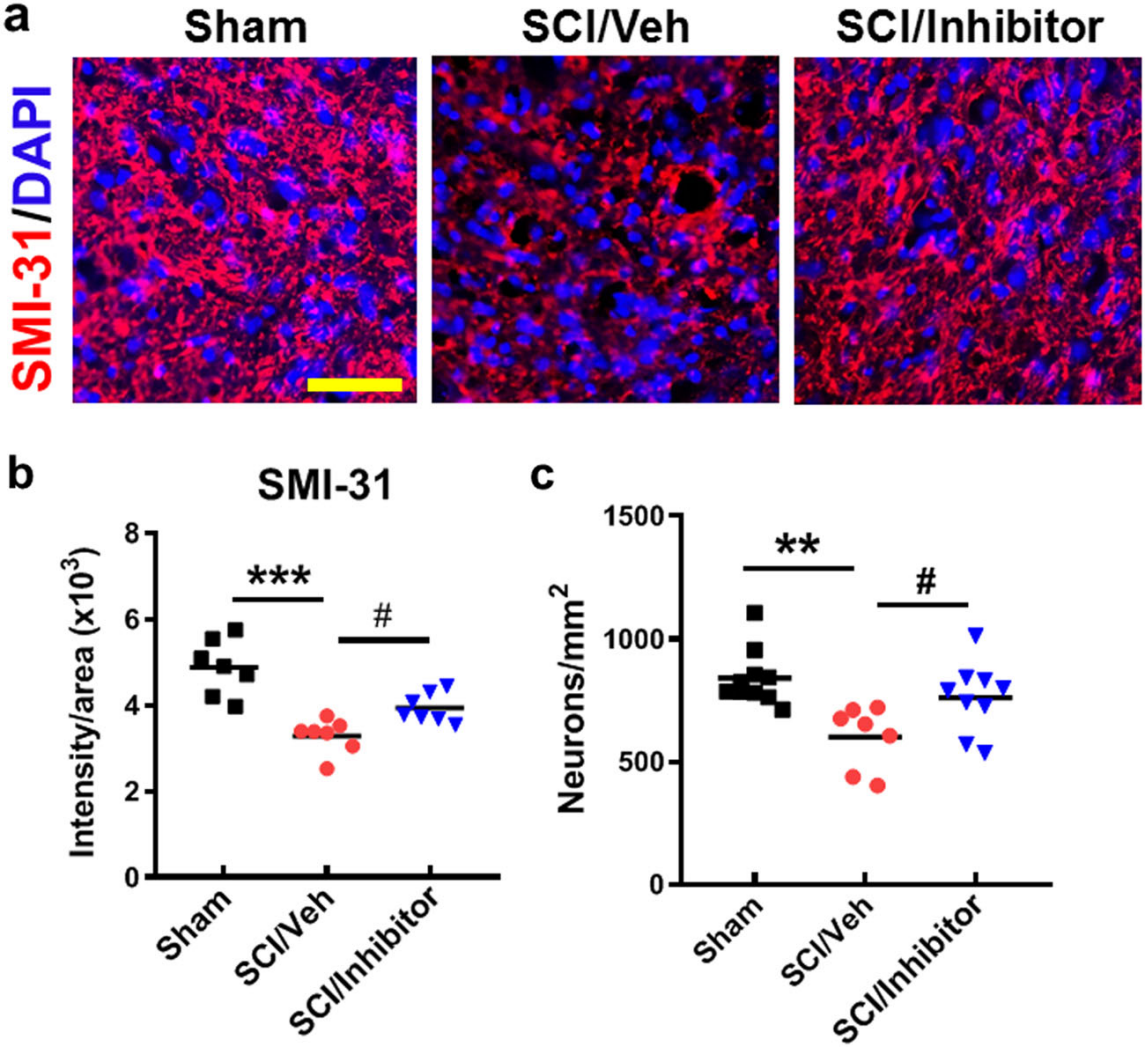
Central administration of miR-711 hairpin inhibitor improves axonal and neuronal survival at 6 weeks after SCI. SCI mice were treated with miR-711 hairpin inhibitors or negative control (vehicle) right after injury followed by an additional treatment at 24 h post-injury. Immunohistochemistry (IHC) staining with axonal marker of phosphorylated neurofilament antibody SMI-31 as well as neuronal marker (NeuN) was performed in the spinal cord coronal sections at 6-week post-injury. **a** Images (×20) of spinal cord sections of Sham, SCI/Veh, and SCI/Inhibitor mice stained with antibodies against SMI-31 (red) and DAPI (blue). Scale bars = 50 μm. **b** Quantification of SMI-31 immunointensity. **c** Quantification of NeuN/DAPI positive cells. The data are presented as independent data points. One-way ANOVA was performed followed by Newman-Keuls multiple comparisons test. *N* = 7/group. **p < 0.01, ***p < 0.01 vs. Sham; ^#^*p* < 0.05 vs. SCI/Veh

To determine whether neuronal damage after SCI is dependent on miR-711 activity, we treated SCI mice with miR-711 hairpin inhibitors or negative control right after injury followed by an additional treatment at 24 h post-injury. Immunohistochemistry (IHC) staining with axonal marker of phosphorylated neurofilament antibody SMI-31 indicated axonal damage or loss in the injured spinal cord at 6-week post-injury (Fig. 3a, b, *p* < 0.01, vs. Sham). Importantly, inhibition of miR-711 significantly increased intensity of SMI-31 + signals (*p* < 0.05, vs. SCI/Veh). Thus, elevated miR-711 appears to contribute to axonal damage in injured spinal cord. Furthermore, NeuN + neurons were also determined at 6 weeks after SCI (Fig. 3c). SCI resulted in 30% of neuronal cell loss, whereas miR-711 inhibitors significantly improved neuronal survival when compared with vehicle-treated samples.

### Ang-1 is rapidly downregulated in the injured spinal cord and reversed by miR-711 hairpin inhibitor

**Fig. 4:**
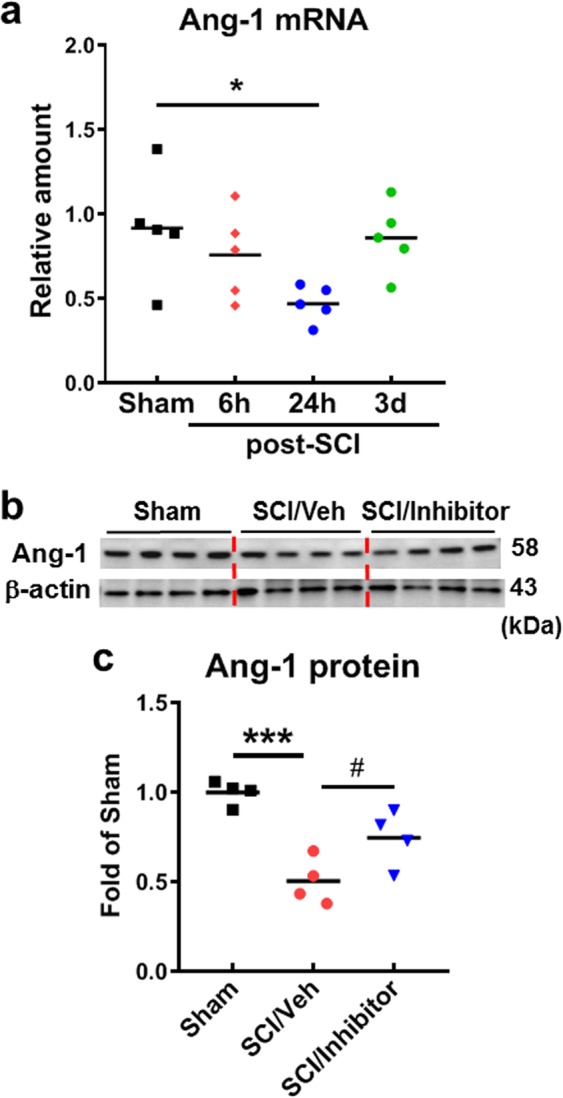
SCI-induced downregulation of Ang-1 expression is significantly attenuated by intrathecal injection of miR-711 inhibitor at day 1 post-injury. **a** Quantification of Ang-1 mRNA by qPCR analysis at indicated time points after injury. *n* = 5–6 mice/group. **b** Expression of Ang-1 protein in the spinal cord tissue surrounding injury site following SCI in mice. β-actin was used as a loading control. **c** Quantification of Ang-1 data from (**b**). *n* = 4 mice/group. The data are presented as independent data points. One-way ANOVA was performed followed by Newman-Keuls multiple comparisons test. *p < 0.05, ***p < 0.001, vs. Sham; ^#^*p* < 0.05 vs. SCI/Veh

To examine effects of SCI on Ang-1 expression, qPCR and western blot analysis were performed in injured spinal cord tissues. There was rapid reduction of Ang-1 mRNA level at 6 h post-injury, with the lowest level at 24 h, and remained low level up to 3 days (Fig. 4a, *n* = 5 mice/group). This was confirmed by western blot at day 1 post-injury (Fig. 4b, *n* = 4 mice/group, *p* < 0.001, vs. Sham). Importantly, intrathecal administration of the miR-711 hairpin inhibitor significantly increased expression of Ang-1 compared with vehicle treatment (Fig. 4c, *p* < 0.05, vs. SCI/Veh), supporting the notion that Ang-1 is a downstream target of miR-711.

### Treatment with miR-711 inhibitor results in significant SCI-induced disinhibition of vascular density

**Fig. 5:**
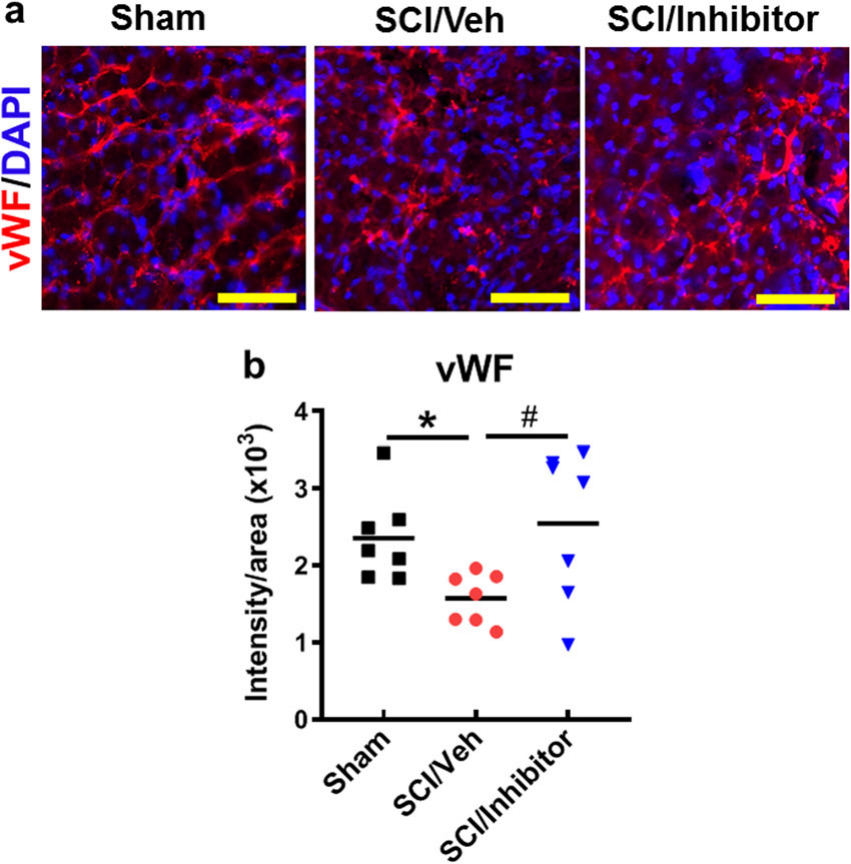
Central administration of miR-711 hairpin inhibitor results in significant SCI-induced disinhibition of vascular density labeled with endothelial marker von Willebrand factor (vWF, red). **a** Representative images from sham or injured spinal cord at 6 weeks post-injury. Scale bars = 50 μm. **b** Quantification of vWF intensity area. The data are presented as independent data points. One-way ANOVA was performed followed by Newman-Keuls multiple comparisons test. *n* = 7/group. *p < 0.01 vs. Sham; ^#^*p* < 0.05 vs. SCI/Veh

To determine effects of miR-711 on the blood vessels, we performed IHC with endothelial marker von Willebrand factor (vWF) that is expressed specifically in the cytoplasm of endothelial cells and that have been used to identify vascular structure after CNS injury^35,36^. We observed that vWF immunointensity was significantly reduced at the injury site 6 weeks post-injury (Fig. 5a), indicating disrupted vascular density. SCI mice treated with miR-711 hairpin inhibitors right after injury followed by an additional treatment at 24 h post-injury showed significant increase of vWF immunointensity when compared with SCI/Veh group (Fig. 5b). This suggests the possibility that elevated miR-711 leads to downregulation of Ang-1, resulting in blood vessels damage and leaky, subsequent detrimental inflammation after SCI.

### Inhibition of miR-711 improves motor function and reduces tissue damage

**Fig. 6:**
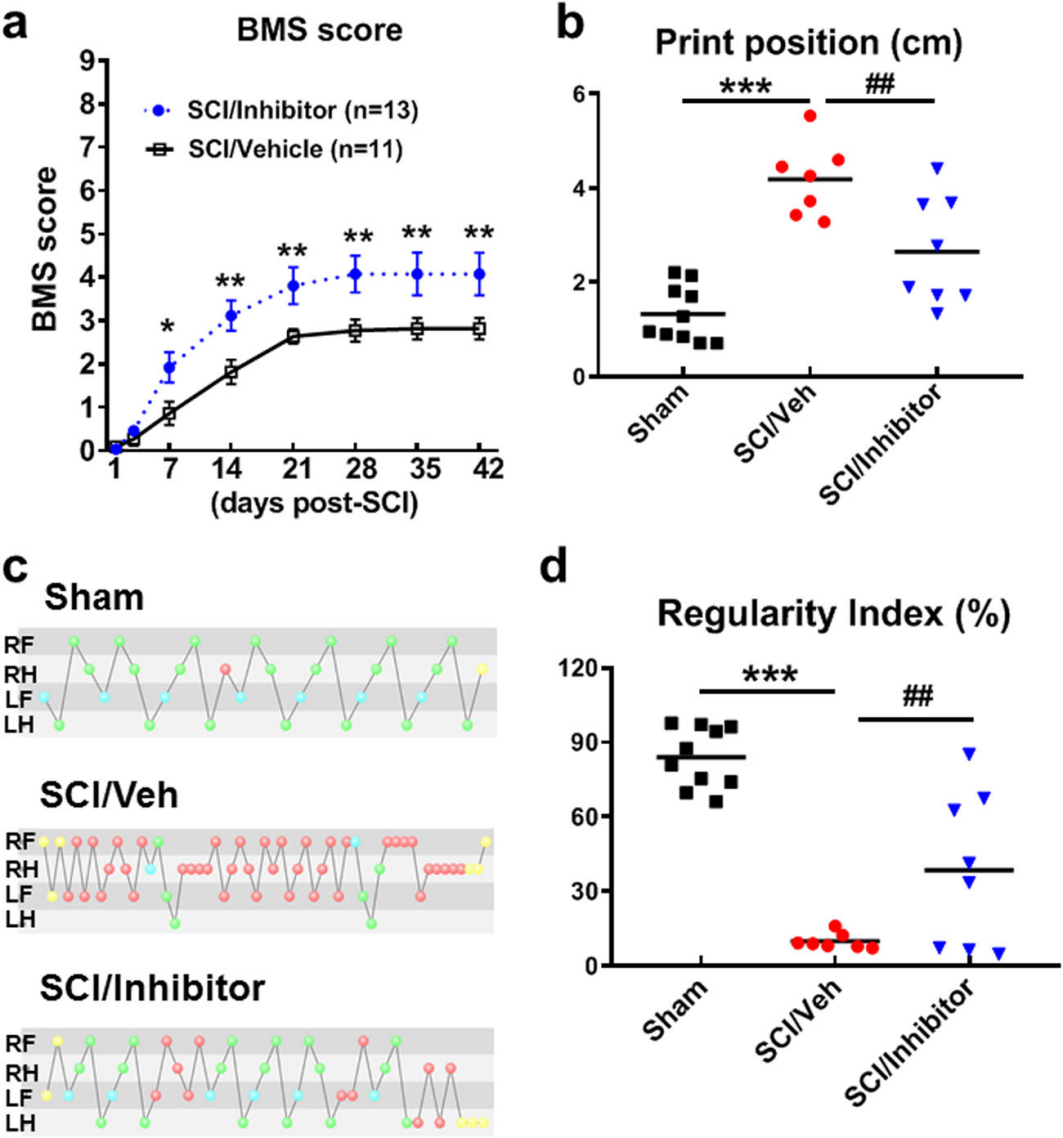
Inhibition of miR-711 improves motor function after SCI. **a** Hindlimbs locomotor function after SCI were evaluated using the Basso Mouse Scale (BMS). The data are presented as mean ± standard error of the mean. Two-way ANOVA with repeated measurements were used followed by Newman-Keuls multiple comparisons test. *n* = 11 (SCI/Veh) and 13 (SCI/Inhibitor). *p < 0.05, **p < 0.01 vs. SCI/Vehicle. **b–d** Motor coordination was evaluated by CatWalk at 5 weeks post-injury. miR-711 hairpin inhibitor treatment attenuated SCI-induced deficits in print position (**b**). Representative step sequences (inter-paw coordination) are indicated in (**c**), where Yellow-not taken into account; Blue-start of a pattern; Green-part of a pattern; Red-not part of a pattern. LH, LR, RH, & RF: left or right hindpaw & forepaw. miR-711 inhibitor-treated mice demonstrated more regular gait patterns than vehicle group (**d**). The data are presented as independent data points. One-way ANOVA was performed followed by Newman-Keuls multiple comparisons test. *n* = 10 (Sham), 7 (SCI/Veh), and 8 (SCI/Inhibitor). ***p < 0.001 vs. Sham/Veh; ^##^*p* < 0.01 vs. SCI/Veh

To assess effects of miR-711 on locomotor functional recovery after SCI, miR-711 hairpin inhibitor-treated and negative control miR-treated SCI mice were tested in the open field on days 1, 3, and weekly up to 6 weeks post-injury. At day 1 after SCI, all mice had a BMS locomotor score of 0 or 1, indicating nearly complete loss of motor function (Fig. 6a). By 1 week after injury, miR-711 inhibitor-treated mice (*n* = 13) had significantly improved BMS scores compared with SCI/Veh animals (*n* = 11, Fig. 6a, *p* < 0.05) which remained through 6 weeks after injury [Post-injury Days: F(7, 176) = 41.021, *p* < 0.001; Drug: F(1, 176) = 35.186, *p* < 0.001; the interaction of Post-injury Days × Drug: F(7, 176) = 1.493; *p* = 0.173; repeated measures two-way ANOVA with Newman-Keuls multiple comparisons test].

Next, more refined quantitative gait analysis by CatWalk was used to detect differences in general motor coordination. At 5 weeks of SCI, the mice that regained adequate locomotor function to be able to withdraw a hindpaw or with plantar position were assessed for CatWalk gait analysis. Print Position is the distance between the position of the hindpaw and the position of the previously placed front paw on the same side of the body and in the same step cycle. A health mouse results in a print position score typically approaching 0–1 cm. SCI (*n* = 7) resulted in significant increase in print positions compared with Sham animals (*n* = 10, Fig. 6b, *p* < 0.001). Remarkably, miR-711 inhibitor-treated mice (*n* = 8) showed significant improvement in print placement compared with SCI/Veh animals (Fig. 6b, *p* < 0.01). To evaluate overall motor coordination, regularity index, a parameter under the category of step sequencing, was selected. Step sequence regularity index expresses the number of normal step sequence patterns relative to the total number of paw placements. It is a fractional measure of inter-paw coordination. In healthy, fully coordinated animals its value is 100%^37^, though in practice, naive mice typically approach 80–90% (Fig. 6c). In response to SCI, the regularity index in SCI/Veh (*n* = 7), which was robustly reduced compared with Sham animals (*n* = 10, Fig. 6d, *p* < 0.001), strikingly appeared to be significantly restored in miR-711 inhibitor-treated mice (*n* = 8, *p* < 0.01), approaching 50% normal stepping. Together, inhibition of miR-711 significantly improves recovery of locomotor function and motor coordination following SCI.

**Fig. 7:**
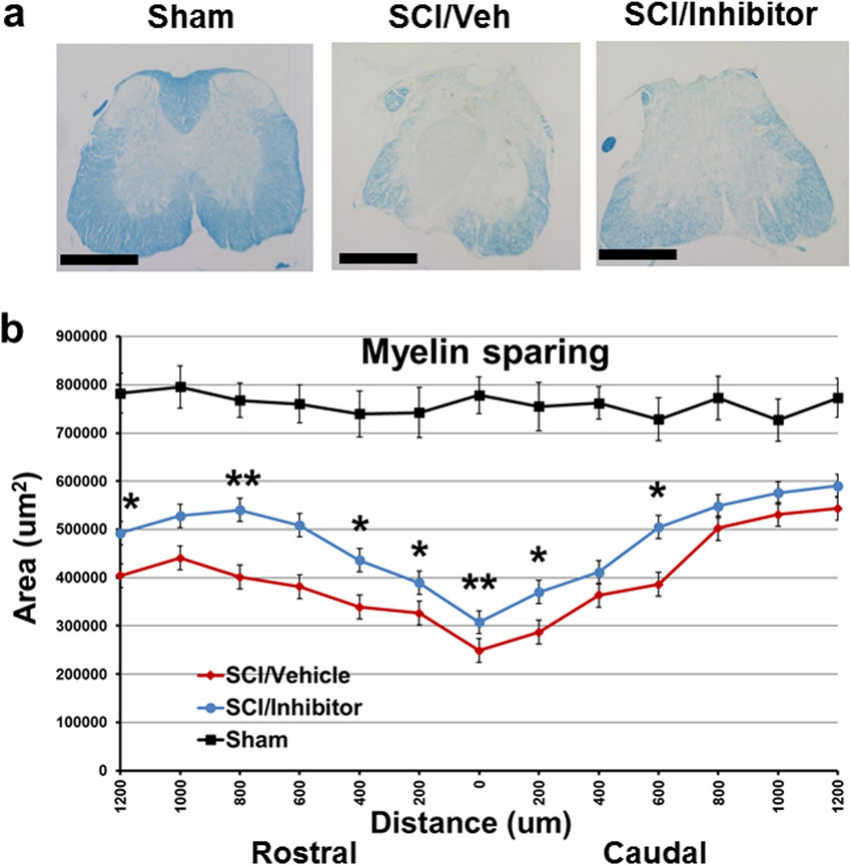
Myelin sparing was examined at various distances rostral and caudal to the injury epicenter using Luxol fast blue (LFB) staining. **a** Representative micrographs showing myelin sparing at the injury epicenter. Images were acquired at ×2.5 magnification. Scale bars = 500 μm. **b** Quantification of spared white matter area rostral and caudal to the lesion epicenter. Student unpaired t tests were performed. *n* = 5–6 mice/group, *p < 0.05, **p < 0.01 vs. SCI/Veh

To determine if the observed behavioral improvement may relate to increased residual white mater, spinal cord sections from injured mice perfused at 6 weeks were stained with LFB for myelinated WM area at 200 μm intervals rostral (R) and caudal (C) to the injury epicenter (EPI). Histological analysis revealed that there was a significant increase of the area of white matter sparing in the miR-711 inhibitor-treated mice compared with SCI/Veh group (Fig. 7). Representative LFB stained sections at the epicenter of each subject illustrate the differences in myelinated WM area between miR-711 inhibitor and the vehicle-treated animals (Fig. 7a). These data indicate that inhibition of miR-711 improves the recovery of motor function and reduces the degree of hyperesthesia after SCI, which is associated with increased WM.

### miR-711 is highly expressed by neurons in vitro

**Fig. 8:**
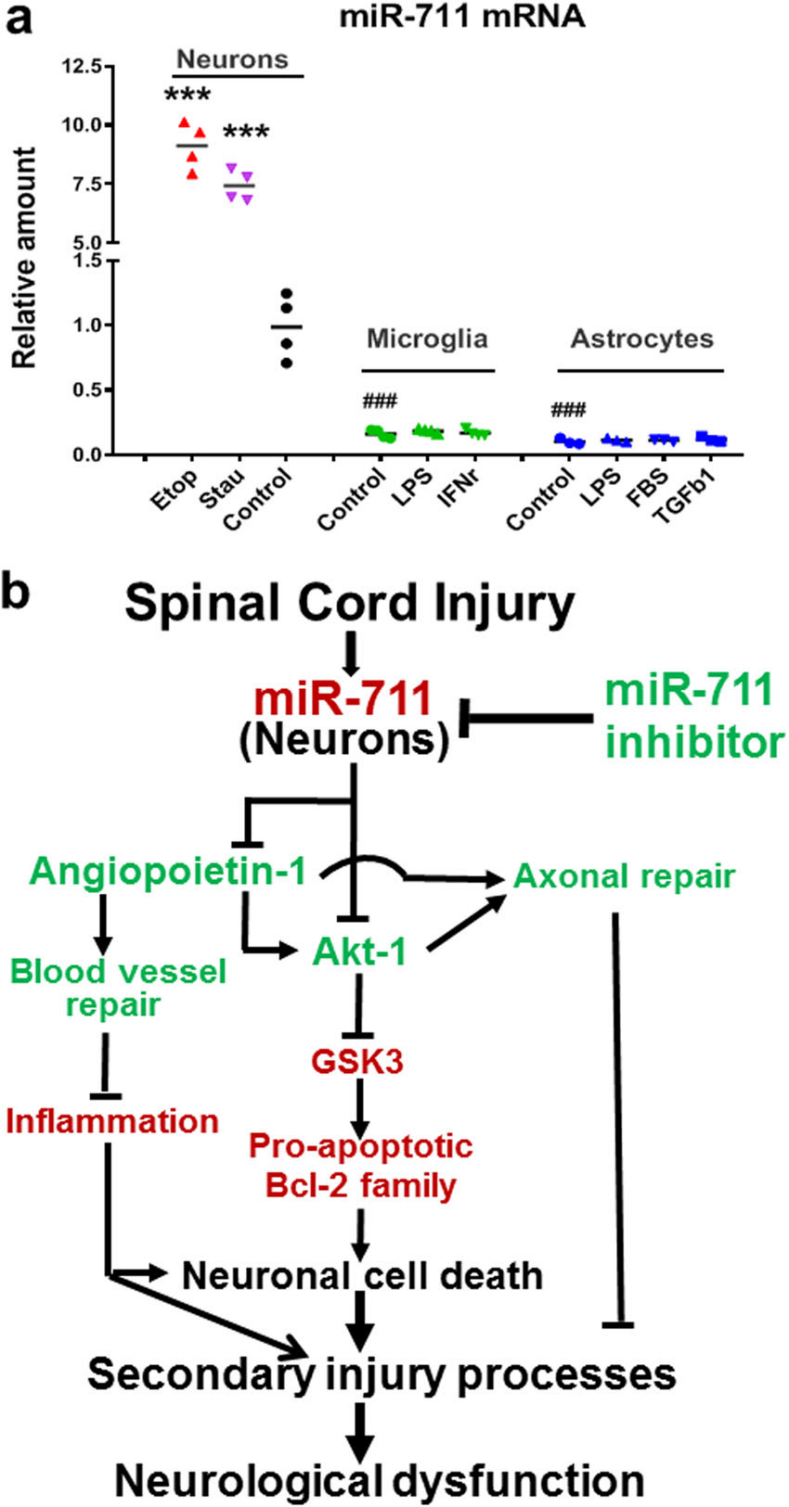
a miR-711 is predominately expressed by neurons in vitro. miR-711 expression was analyzed by qPCR in primary cultured cortical neurons, microglia, and astrocytes. Neurons were exposed to cell death inducers etoposide (Etop, 25 nM) or staurosporine (Stau, 25 nM) for 6 h, microglia were treated with LPS (30 ng/ml) or IFNγ (60 ng/ml) for 24 h, astrocytes were treated with LPS (1 μg/ml), TGFβ1 (30 ng/ml), or 10% FBS for 24 h. The data are presented as independent data points. One-way ANOVA was conducted followed by Newman-Keuls multiple comparisons test. *n* = 3–5 dishes/group repeated in three independent cultures. ***p < 0.001, ^###^*p* < 0.001 vs. Control (neurons). **b** The functional role of miR-711 in regulating neuronal cell death, axonal and blood vessel damage after SCI. SCI-induced elevation of miR-711 can initiate secondary injury pathways including neuronal cell death, axonal damage, and vascular disruption, through in part, inhibition of Akt and Angiopoietin-1 (Ang-1) pathways. Administration of miR-711 inhibitor in the acute period after SCI has significant neuroprotective effects, likely acting both directly on neurons and by limiting Ang-1-mediated vascular permeability effects

miR-711 level has been shown to be rapidly elevated by neuronal insults in vitro^33^. To compare miR-711 expression levels in CNS different cell types, primary neurons, microglia, and astrocytes were cultured from rat embryonic or neonatal cortices. Primary cortical neurons were incubated with cell death inducers etoposide (Etop) or staurosporine (Stau) for 6 h. In consistent with our previous report^33^, Etop or Stau-induced neuron apoptosis resulted in significant elevation in levels of miR-711 compared with control neurons (*n* = 3–5 dishes, Fig. 8a, *p* < 0.001). Primary microglia were exposed to lipopolysaccharide (LPS) or interferon γ (IFNγ) for 24 h, separately. To activate astrocytes, LPS, transforming growth factor β1 (TGFβ1), or 10% fetal bovine serum (FBS) were applied to the cultures, separately. After 24 h treatment, the cells were harvested for RNA extraction and miR-711 analysis. We found extremely lower basal level of miR-711 in primary cultured astrocytes and microglia than that in cultured cortical neurons (*p* < 0.001). Moreover, activated microglia and astrocytes failed to stimulate miR-711 expression (Fig. 8a). These data suggest that miR-711 is predominately expressed by neurons.

## Discussion

In order to identify “master regulators” of post-traumatic apoptosis, we initially focused on miR changes in the first hours to days after SCI, a period associated with maximal secondary neuronal cell death. Among miRs showing highly significant changes early after traumatic injury in vivo was miR-711, which was markedly (9–10-fold) and rapidly (6 h post-injury) upregulated. We also focused on miR-711 because it was predicted to target several key regulatory factors including Akt and Ang-1. In addition, miR-711 expression is robustly upregulated for days in injured brain after TBI as well as in the heart after ischemia/reperfusion insult^20,33^. Our results show that SCI induces elevation of miR-711 expression and reduction of its targets Akt and Ang-1 as early as 6 h post-injury. Blocking miR-711 activation by using miRIDIAN Hairpin Inhibitors limits SCI-mediated Akt/GSK3β and Ang-1 inactivation, leading to neuroprotective effects. Importantly, acute miR-711 inhibition significantly reserved spared white matter and improved behavioral recovery after SCI.

Neurotrauma-mediated dysregulated Akt expression has been controversial. Some studies reported rapidly decreased Akt levels^33,38–41^, whereas others showed increased levels of total and phosphorylated Akt^42,43^. We found that Akt is robustly downregulated in the injured spinal cord after moderate contusive SCI. This reduction, including both the phosphorylated form and total Akt, suggests a pathophysiological role for Akt dysregulation, consistent with our previous report in a mouse TBI model^17,33^. These divergent reports may reflect different lesion paradigms and tissue sampling or time windows after injury. Nonetheless, endogenous activation of Akt in neurons may represent a neuroprotective response to injury. Ambacher et al.^44^ reported that Akt-dependent phosphorylation on Ser9 is a key mechanism that inactivates GSK3β. It is known that GSK3β plays a pro-apoptotic role in several models of neuronal cell death: inhibition of GSK3β promotes neuronal survival, whereas overexpression causes neuronal apoptosis^44^. Active/dephosphorylated GSK3β induces PUMA expression by modulating FoxO3a activity, leading to neuronal cell death. We reported that inhibition of miR-711 partially restores the levels of total and phosphorylated/active Akt, as well as the levels of phosphorylated/inactive GSK3β after SCI. These alterations are correlated with reduced PUMA expression and cleavage fragments of α-fodrin in the spinal cord after injury. Interventions preventing post-traumatic Akt inactivation can improve functional and histological outcomes after SCI^21,40,45,46^. miR-21 targets PTEN, an Akt inhibitor and the neuroprotective effects of miR-21 mimics after TBI may reflect activation of the Akt pathway^36,47^. In the present study, inhibition of miR-711 partially rescued the Akt/GSK3β signaling; this was associated with reduced neuronal cell death and axonal damage, as well as improved functional recovery.

Angiopoietin-1 (Ang-1) is recognized as an endothelial growth factor. Studies demonstrate that Ang-1 signal through the Tie2 receptor promotes endothelial cell survival, stabilizes blood vessels and reduces leakiness during developmental or tumor-associated angiogenesis^48–50^. Importantly, Ang-1 is another predicted target for miR-711. We recently identified^28^ that miR-711 directly targets the Ang-1 messenger RNA, decreasing Ang-1 expression. We also observed short-term neuroprotective effects after Ang-1 administration after TBI. Previously, the neuroprotective effect of Vasculotide (Ang-1 Mimetic Peptide) was demonstrated in vivo in models of experimental stroke^29^: intraperitoneal injections with Vasculotide attenuated caspase activation and apoptosis. In SCI, an Ang-1 mimetic rescues epicenter blood vessels through its pro-survival role for endothelial cells, resulting in reduced inflammation, and improved white matter and locomotor function^51,52^. Ang-1 is also expressed in CNS neurons and its neuroprotective role may involve, in part, Akt activation^29^. In the present study, we showed that SCI induced a rapid downregulation of Ang-1 in the injured spinal cord that was sustained over several days. Inhibition of miR-711 rescued epicenter blood vessels associated with elevation of Ang-1. Thus, we speculate that promoting the endothelial-selective Ang-1 by miR-711 inhibitors may provide vascular protection and reduced inflammation, thereby improving functional outcomes. Whether or not miR-711 needs Ang-1 to function efficiently is intriguing for future investigation.

Increasing evidence suggests that miRNAs represent a new class of drug targets^53–57^, with several miRNAs evaluated both preclinically and clinically^56–58^. miRIDIAN microRNA Hairpin Inhibitors are the newest generation proprietary, synthetic inhibitors targeting all human, mouse, and rat microRNAs in the miRBase database release 21.0. miRIDIAN Hairpin Inhibitors are single-stranded, chemically enhanced RNA oligonucleotides designed to bind and to sequester the complimentary, mature microRNA strand. This design allows for microRNA inhibition to be maintained longer than with other available synthetic inhibitors. Importantly, enhanced potency and longevity allow for multiplexed microRNA inhibition at very low nanomolar concentrations and with minimal toxicity, which reduces the risk of negative and off-target side effects. We have successfully applied miRIDIAN microRNAs in a TBI model^17,33^ as well as in a SCI model here. The specificity of highly potent miRNA inhibitors has been tested (gelifesciences.com/dharmacon). They do not cross-react with non-family member miRNAs that share sequence similarity. As miR-711 does not have any family members, thus it is not a concern regarding the specificity of miR-711 inhibitor in suppressing miR-711 and whether the inhibitors may also have direct effects on the expression of other miRNAs.

Effects of miR-711 on cell proliferation have been controversial in cancer cell lines. In gastric cancer cell line, upregulated expression of miR-711 arrests the cells in the G1 phase by downregulating expression of cyclin-dependent kinases 4 (CDK4)^59^, suggesting a participation of miR-711 signaling in cell cycle regulation. However, downregulating miR‑711 inhibits cell proliferation, colony formation, migration and invasion and enhances the rate of apoptosis of breast cancer cells^60^. After SCI, a key pathophysiological mechanism appears to be cell cycle activation (CCA)^61^. Experimental evidence in both SCI and TBI models, much from our laboratory, indicates that in post-mitotic cells such as neurons and mature oligodendroglia, CCA occurs post-trauma and contributes to neuronal cell death, whereas in microglia or astrocytes it causes proliferation and activation^61^. Although elevated miR-711 in gastric cancer cells showed suppression of CDK4^59^, cell cycle machinery is a complicated process in vivo. Moreover, our data indicate that inhibition of miR-711 reduces neuronal cell death in vitro^33^ and in vivo after SCI, associated with reduced CCA. Whether or not elevated CCA in the injured spinal cord is suppressed by miR-711 inhibition remains to be determined.

miRNAs are implicated in the pathophysiology of CNS disorders but the underlying mechanisms have been difficult to study in part due to the cellular heterogeneity in vivo. miR-711 levels are rapidly elevated by neuronal insults in vitro^33^. We found significant lower level of miR-711 in primary cultured astrocytes and microglia than that in cultured cortical neurons. LPS, 10% FBS, or TGFβ1 are known activate astrocytes but failed to stimulate miR-711 expression. Moreover, miR-711 levels in microglia remain unchanged in response to LPS or IFNγ, consistent with a previous report^62^. These data suggest that miR-711 is predominately expressed by neurons. However, miR-711 was reported to be downregulated in M2a-skewed microglia stimulation (IL-4)^62^, suggesting that reduced expression in miR-711 may regulate anti-inflammatory effects. Future studies should further examine cell specific miR-711 expression changes and its validated predicted targets (Akt and Ang-1) after CNS injuries.

Recent studies^63,64^ have shown that intravenous administration of microRNA inhibitors are effective in models of focal cerebral ischemia; the microRNA inhibitors reached the central nervous system and levels of the targeted microRNA in the brain were significantly reduced as early as 2 h after a single iv injection and remained low for at least 24 h^63^. Whether or not systemic treatment of miR-711 inhibitor is effective in spinal cord injury remains to be determined.

Gender-related differences after SCI have been reported with regard to neurologic changes and post-traumatic neuropathic pain^65–68^. Expression levels of miR-711 and Ang-1 in response to SCI were compared between male and female mice. No differences of miR-711 mRNA or Ang-1 mRNA were observed between the sexes (data not shown). However, whether miR-711 inhibitor-treatment effects differ between female and male mice should be examined in future studies.

In summary (Fig. 8b), we provide evidence that SCI induces an elevation of miR-711 expression and reduction of its targets Akt and Ang-1, which is correlated with neuronal cell death and axonal damage. Inhibition of miR-711 by miRIDIAN microRNA Hairpin Inhibitors significantly rescues the Akt/GSK3β signaling as well as Ang-1 expression after SCI, associated with reduced neuronal/axonal damage and protection of epicenter blood vessels. Administration of miR-711 inhibitor in the acute period after SCI has significant neuroprotective effects, likely acting both directly on neurons and by limiting Ang-1-mediated vascular permeability effects. Treatment was associated with reduced tissue damage and marked improvement in functional recovery.

## Materials and methods

### Animals and contusive spinal cord injury

All experiments were conducted using adult male C57BL/6 mice (10–12 weeks, 20–25 g, purchased from Taconic, Rensselaer, NY). The animals were housed on a 12:12 h light/dark cycle with food and water freely available ad libitum. All procedures were carried out under protocols approved by the Institutional Animal Care and Use Committee (IACUC) at the University of Maryland School of Medicine. A moderate spinal cord contusion injury was induced using the Infinite Horizon (Precision Systems and Instrumentation) spinal cord impactor as previously described^69–72^. Briefly, mice were deeply anesthetized with isoflurane evaporated in a gas mixture containing 70% N_2_O and 30% O_2_ and administered through a nose mask (induction at 3% and maintenance at 1.5%). The spinal column was stabilized using lateral clamps over the lateral processes at T9 and T11. A laminectomy was performed followed by a midline spinal contusions at T10 level with a force of 60 kdyn for 2 s dwell time, a moderate injury. Control sham animals received laminectomy only. The bladders of injured mice were manually expressed 2–3 times per day until a reflex bladder emptying was established. After SCI, all mice were assigned to a treatment group according to a randomized block experimental design. Individuals performed functional assessment and involved in data analysis were blinded to group designations throughout all stages of the experiment. The number of mice at various time points in each study is indicated in the figure legends. All mice were assessed at various time points after injury by the same surgeon and at the same period of time.

### Intrathecal injection of miR-711 hairpin inhibitor

miR-711 hairpin inhibitors (miRIDIAN microRNA mouse mmu-miR-711 hairpin inhibitor, IH-310774-03-0005) or negative controls (Vehicle, miRIDIAN microRNA hairpin inhibitor negative control; IN-001005-01-05) were purchased from GE Healthcare Dharmacon Inc. and reconstituted in artificial cerebrospinal fluid (aCSF). Right after injury, the bilateral microinjections (2 injections/side, 1.25 μl/injection, total 5 μl) of the miR-711 inhibitor or Vehicle (final concentration 0.5 nmol/mouse) were made intrathecally at 1 mm from the midline^34^. There was a 2 mm distance between the two injections on each side. The dose of the miR-711 inhibitor was based on the results obtained from in vitro cultured neurons and mouse TBI model^33^. Based on our pilot data showing no effects of miR-711 inhibitor in sham mice, Sham/Vehicle group served as controls. Animals were euthanized at 24 h following trauma for biochemical analysis.

As miR-711 expression levels show sustained elevation for several days, we also applied the additional treatment at 24 h post-injury for long-term behavioral tests, in which direct percutaneous intrathecal injections were made by lumbar puncture^72^. Briefly, mice were anesthetized with isoflurane and the pelvis was stabilized to allow the identification of the space between the L5 and L6 spinous processes. A ½ inch 30 g needle attached to a 25 μl Hamilton syringe was percutaneously inserted into the groove between the spinous and transverse processes of the L5 and L6 vertebrae at a 20° angle in the rostral direction. Entry into the intrathecal space was determined by visualizing a quick tail flick during needle penetration, at which point the needle angle was decreased to 10°. miR-711 inhibitor (0.5 nmol in 5 μl aCSF per mouse) or the same amount of Vehicle were administered by intrathecal injection. The mice recovered under a warming lamp and were observed for signs of spinal trauma (hindlimb weakness, unsteady gait, dragging a hindpaw) beyond what is present due to the experimental SCI. Any mice with signs of spinal trauma were euthanized immediately.

### RNA isolation and quantitative polymerase chain reaction (qPCR) analysis

Total RNA was isolated by using a miRNeasy isolation kit (QIAGEN) with on-column DNase treatment (QIAGEN). During the process of isolation, samples were treated with RNase-free DNase (Qiagen) to prevent DNA contamination of the samples according to the manufacturer’s protocol. Verso cDNA Kit (Thermo Scientific) was used to synthesize cDNA from purified total RNA. RNA (1 μg) was heated to 70 °C for 5 min and mixed with 5X cDNA-synthesis buffer, dNTP mix (0.5 nM final concentration), Verso Enzyme Mix, and random hexamers (400 ng/μl) were added. Tubes were incubated at 42 °C for 30 min, followed by 95 °C for 2 min. Quantitative real-time PCR amplification was performed by using cDNA TaqMan Universal Master Mix II (Applied Biosystems). Briefly, reactions were performed in duplicate containing 10 µl of 2 X TaqMan Universal Master Mix II, 2 μl of cDNA (corresponding to 50 ng RNA/reaction) and 1 µl of 20 X TaqMan Gene Expression assay primers in a final volume of 20 μl. TaqMan Gene Expression assays for following: GAPDH (Mm99999915_g1), Ang-1 (Mm00456503_m1) were used. Reactions were amplified and quantified using the QuantStudio 5 Real-Time PCR System and the corresponding software (Applied Biosystems). The PCR profile consisted of one cycle at 50 °C for 2 min and 95 °C for 10 min, followed by 40 cycles of 95 °C for 15 s and 60 °C for 1 min. The efficiency of reactions was measured using the CT slope method. Gene expression was normalized to GAPDH, and the relative quantity of mRNAs was calculated based on the comparative CT method^73^. Analysis of relative gene expression data using real-time quantitative PCR and the 2(-Delta Delta C(T)) method.

### miR reverse transcription and miR-711 qPCR analysis

qPCR was used to measure the expression of mature miR-711. Ten nanograms of total RNA was reverse transcribed using TaqMan miRNA Reverse Transcription Kit (Applied Biosystems) with miRNA-specific primers. Reverse transcription reaction products (1.5 μl) were used for qPCR as described above. TaqMan Gene Expression assays for mmu-miR-711 (001646) and U6 snRNA (001973) (Applied Biosystems) were used. miR-711 expression was normalized to U6 snRNA.

### Western blots

Mouse spinal cord tissue (approximately three-millimeter segments) centered on the injury site was harvested at indicated times after injury. Western blot analysis was performed as described previously^72^. Briefly, the tissues were homogenized in RIPA buffer (Sigma-Aldrich) supplemented 1x protease inhibitor cocktail (Sigma-Aldrich), phosphatase inhibitor cocktails II and III (Sigma-Aldrich), sonicated and centrifuged at 20,000 × *g* for 20 min. Protein concentrations were determined by the Pierce BCA method (ThermoFisher Scientific, USA). Samples were run on 4–20% SDS-PAGE (Bio-Rad, Hercules, CA), and transferred to nitrocellulose membrane (Bio-Rad). Primary antibodies included: mouse anti-phospho Akt serine 473 (p-Akt; 1:500, Cat# 4060, Cell signaling Technology, Inc., San Diego, CA), mouse anti-phospho Akt threonine 308 (p-Akt; 1:500, Cat# 9275, Cell signaling Technology), mouse anti-Akt (pan Akt, 11E7, 1:1000; Cat# 4685, Cell signaling Technology, Inc.), rabbit anti-phospho-GSK3 α/β (Ser21/9, p-GSK3, 1:1000; Cat# 9331, Cell signaling Technology, Inc.), rabbit anti-GSK3α/β (D75D3, GSK3α/β, 1:1000; Cat# 5676, Cell signaling Technology, Inc.), rabbit anti-PUMA (1:1000; Cat# 3041, ProSci Incorporated), mouse anti-α-fodrin (1:5000; Cat# BML-FG6090, Enzo Life Sciences), Angiopoietin-1 (1:1000; Cat# GTX28451, GeneTex Inc.), and mouse anti-β-actin (1:5000; Cat# AB2302, Millipore, Temecula, CA). Immune complexes were detected with the appropriate HRP conjugated secondary antibodies (KPL, Inc., Gaithersburg, MD) and visualized using SuperSignal West Dura Extended Duration Substrate (Thermo Scientific, Rockford, IL). Chemiluminescence was captured on a Kodak Image Station 4000 R station (Carestream Health Inc., Rochester, NY) and protein bands were quantified by densitometric analysis using Carestream Molecular Imaging Software (Carestream Health Inc., Rochester, NY). The data presented reflects the intensity of target protein band compared with control and normalized based on the intensity of the endogenous control for each sample (expressed in fold of sham).

### Behavioral assessments

#### Locomotor testing (BMS score)

All behavioral tests were blindly performed. Mice were tested for hindlimb function in open-field locomotion on day 1 after injury and weekly thereafter for up to 6 weeks using the Basso mouse scale (BMS) for locomotion^74^. Briefly, mice were placed in an open-field chamber (diameter = 40 in) and observed for 4 min by two trained observers. Animals were rated on a scale of 0–9: 0 being complete paralysis, and 9 being normal locomotion based on hind limb joint movement, weight support, plantar stepping, and coordination. A minimum score of 4, or occasional plantar stepping, was required to evaluate each animal in additional measures for pain-like behaviors as well as more sensitive measures of motor and coordinative recovery.

#### CatwalkXT auto mated gait analysis

Gait analysis was performed using the CatwalkXT automated system^69^ (Noldus; RRID:SCR_004074). Each mouse underwent only one testing session at 5 weeks after SCI to maintain situational novelty and encourage exploration of the CatWalk. Data acquisition took place in a darkened room with the same researcher handling each subject. The CatWalk itself features a red overhead lamp and green illuminated walkway, which responds to the pressure of the animals’ weights and obtains live foot print videos. Animals were first placed in the open end of the CatWalk under the red ceiling light and allowed to walk across the walkway to the darkened escape enclosure. A minimum of three valid runs, or complete walkway crossings, were obtained for each subject. Trials in which the animal stopped partway across or turned around during a run were excluded from analysis. The primary variables were print position (the distance between the hind and forepaw of one side during gait) for motor coordination, and regularity index (the percentage of step cycles that can be characterized as fitting a standard pattern of gait).

#### Tissue processing and assessment of white matter sparing

Following animal perfusion with 4% paraformaldehyde, spinal cord segments containing the lesion area were dissected out, embedded, and cut into 20-μm-thick serial sections placed serially on set of 10 slides for 10 sets of slides. A representative slide from each set was then stained for myelin using Luxol fast blue (LFB) to determine the location of the lesion epicenter, defined as the section with the least amount of spared white matter (WM). Residual WM was also calculated for areas rostral and caudal to the lesion epicenter^72^. Images were captured at ×2.5 magnification and analyzed using National Institutes of Health ImageJ software (RRID:SCR_003070). The threshold level of each 8 bit image was set to mark only LFB-positive tissue, and total LFB-positive area was calculated for each section.

#### Immunohistochemistry, image acquisition, and quantification

Immunofluorescence staining was followed procedures described previously^69,72^. Briefly, slides were first washed gently in PBS, dried, and placed in a vacuum chamber for 20 min to promote adhesion. Tissue was then blocked in 5% normal goat serum for 1 h, and incubated overnight at 4 °C in primary antibodies, including PhosphoDetect™ Anti-Neurofilament-H Mouse mAb (SMI-31, 1:1000; Sigma-Aldrich, NE1022), NeuN (1:500; Millipore, MAB377), and von Willebrand factor (vWF, 1:1000; Millipore, AB7356). The next day, slides were washed and incubated for 2 h in fluorescent secondaries (Alexa fluor), and washed again. Then DAPI nuclear stain (Sigma-Aldrich) was added for 30 min before a final wash, followed by cover slipping in non-fluorescing mounting medium (National Diagnostics, HS-106). All immunohistological staining experiments were performed with appropriate positive control tissue as well as primary/secondary-only negative controls.

For quantitative image analysis, images were acquired using a fluorescent Nikon Ti-E inverted microscope, at 20× (CFI Plan APO VC 20×; numerical aperture, 0.75; working distance, 1 mm) magnification and quantified using NIS-Elements AR software (Nikon). Exposure times were kept constant for all sections in each experiment. Background for all images was subtracted using Elements. All images were quantified using Elements: nuclei were identified using Spot Detection algorithm based on DAPI staining; cells positive for any of the immunofluorescence markers were identified using Detect Regional Maxima or Detect Peak salgorithms, followed by global thresholding. The number or immunointensity of positive cells was normalized to the total area imaged. The immunointensity of SMI-31 positive signals were acquired from both sides of dorsal, ventral, and lateral whiter matter. The number of NeuN labeled cells were acquired at rostral or caudal to the epicenter with total # from both sides of ventral horns (VH), dorsal horns (DH), and intermediate gray matter. To assess effects of damaged neurons on the blood vessels, the immunointensity of vascular density were acquired from the grey matter. All images were acquired 2–3 mm rostral or caudal to the epicenter, with *n* = 15–20 images per location from 10 to 15 sections per mouse. All images were captured from *n* *=* 7–9 mice per group. For each experiment, data from all images from one region in each mouse were summed up and used for final statistical analysis. At least 1000–2000 cells were quantified per mouse per area per experiment^75,76^. The images were processed using Adobe Photoshop7.0 software (Adobe Systems).

### Primary neurons, microglia, and astrocytes culture

Primary neuronal cultures were derived from E18 rat cerebral cortices, as previously described^77^. Cells were seeded onto poly-D-lysine-coated (50 μg/ml, 70–150 kDa, Sigma-Aldrich, Cat# P6407) 10 cm Petri dishes (cell density 1 × 10^6^/cm^2^) and maintained in serum-free conditions using Neurobasal medium supplemented with 2% B27, 25 mM Naglutamate, and 0.5 mM L-glutamine. At 7 days after the culture, neurons were exposed to cell death inducers etoposide (Etop, 25 nM) or staurosporine (Stau, 25 nM) for 6 h,

Primary mixed glia were cultured from the cerebral cortex of neonatal rats as described^77^. The cells were plated in T75 flasks and grown in Dulbecco’s modified Eagle’s medium (DMEM)/F12 supplemented with 10% FBS, 1% Pen/Strep at 37 °C with 5% CO_2_. When the cells had grown to confluence, the flasks were shaken at 100 rpm for 1 h at 37 °C and the supernatant contained enriched microglia. The flasks were continuously shaken for 2–3 days, the remaining cells were enriched astrocytes. The yield microglia and astrocytes were re-plated in 3.5 or 6 cm dishes. When microglia had reached confluence, LPS (30 ng/ml) or IFNγ (60 ng/ml) were applied to the dish for 24 h. The astrocytes were treated with LPS (1 μg/ml), TGFβ1 (30 ng/ml), or 10% FBS for 24 h. The cell lysates were harvested for RNA extraction and miR-711 qPCR analysis.

### Statistical analysis

Quantitative data were plotted as mean ± standard error of the mean or independent data points. The number of animals in all studies was determined by power analysis (power of 0.8 with alpha value 0.05). For the BMS scores, repeated measures two-way analyses of variance (ANOVAs) were conducted, followed by Newman-Keuls multiple comparisons test to compare the differences between groups. Statistical significance was evaluated between two individual samples using Student unpaired *t* tests. For multiple comparisons, one-way analysis of variance (ANOVA) followed by Newman-Keuls multiple comparisons test. Statistical analysis was performed using SigmaPlot Program, Version 12 (Systat Software) or GraphPad Prism software, version 4.00 for windows (GraphPad Software, Inc). A *p* value of <0.05 was considered statistically significant.

## Supplementary information

Sup Material

Authorship

Checklist
