# Supplementary material for: Inhibition of microRNA-711 limits angiopoietin-1 and Akt changes, tissue damage, and motor dysfunction after contusive spinal cord injury in mice
Source: Cell Death Dis. 2019 Nov 4;10(11):839. doi: 10.1038/s41419-019-2079-y (PMC6828685; doi:10.1038/s41419-019-2079-y)
Supplement: Supplementary file 1 — Sup Material [file 41419_2019_2079_MOESM1_ESM.pdf]

Supplementary Information:

- Author Contribution Form (PDF)
- A Reproducibility Checklist (PDF)
