# Supplementary material for: Inhibition of microRNA-711 limits angiopoietin-1 and Akt changes, tissue damage, and motor dysfunction after contusive spinal cord injury in mice
Source: Cell Death Dis. 2019 Nov 4;10(11):839. doi: 10.1038/s41419-019-2079-y (PMC6828685; doi:10.1038/s41419-019-2079-y)
Supplement: Supplementary file 2 — Authorship [file 41419_2019_2079_MOESM2_ESM.pdf]

DECLARATION OF CONTRIBUTIONS TO ARTICLE

ADMC

Manuscript Number: CDDIS-19-3272

Journal Name: *Cell Death & Disease* (the 'Journal')

Proposed Title of the Contribution:

Inhibition of microRNA-711 limits angiopoietin-1 and Akt changes, tissue damage, and motor dysfunction after contusive spinal cord injury in mice (the 'Contribution)

Author(s):

Boris Sabirzhanov1,*, Jessica Matyas1,*, Marina Coll-Miro1, Laina Lijia Yu1, Alan I. Faden1,2, Bogdan A. Stoica1, and Junfang Wu1,2,# (the 'Authors')

For all *CDDis* articles, each person named as an author in the published version must be able to show he or she has contributed substantially to the article.

Authorship credit should be based on 1) substantial contributions to conception and design, acquisition of data, or analysis and interpretation of data; 2) drafting the article or revising it critically for important intellectual content; and 3) final approval of the version to be published. Authors should meet conditions 1, 2 and 3.

Any person who cannot be shown to have made a substantial contribution to the article cannot be listed as an author in the final version. The name of any person who is deemed to have made a minor contribution can, however, appear in the Acknowledgments section of the article.

Please complete the table below to indicate the contributions of all named authors to the manuscript.

Author Full Name:	Specification of Contribution to the Manuscript:
Boris Sabirzhanov	designed experiments, performed experiments, analyzed data, prepared figures, revised the manuscript
Jessica Matyas	designed experiments, performed experiments, analyzed data, prepared figures, commented on the manuscript
Marina Coll-Miro	performed experiments, analyzed data, prepared figures, commented on the manuscript
Laina Lijia Yu	performed experiments, analyzed data, commented on the manuscript
Alan I. Faden	conceived the project, interpreted results, revised the manuscript
Bogdan A. Stoica	conceived the project, designed experiments, interpreted results, revised the manuscript
Junfang Wu	conceived the project, designed experiments, performed experiments, analyzed data, interpreted results, prepared figures, wrote the manuscript

Please complete the table below to indicate the contributions of all named authors to the figures.

Figure 1:

BS and JW performed experiments, analyzed data, and prepared figure.

Figure 2:

BS and JW performed experiments, analyzed data, and prepared figures.

Figure 3:

JM, MCM, LLY and JW performed experiments and analyzed data, JW prepared figures.

Figure 4:

BS, MCM, and JW performed experiments, BS and MCM analyzed data, BS and JW prepared figures.

Figure 5:

JM, MCM, LLY, and JW performed experiments and analyzed data, JW prepared figures.

Figure 6:

JM, LLY, and JW performed experiments and analyzed data, JM and JW prepared figures.
Figure 7: MCM, LLY, and JW performed experiments and analyzed data, MCM and JW prepared figures.
Figure 8: BS and JW performed experiments, BS analyzed data, and BS, BAS, and JW prepared figure.

Signed for and on behalf of the Author(s):

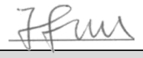

Print Name: Junfang Wu

Date: 10-10-2019
